# Characterization of a novel cysteine-less Cu/Zn-superoxide dismutase in *Paenibacillus lautus* missing a conserved disulfide bond

**DOI:** 10.1016/j.jbc.2023.105040

**Published:** 2023-07-11

**Authors:** Yoshiaki Furukawa, Atsuko Shintani, Shuhei Narikiyo, Kaori Sue, Masato Akutsu, Norifumi Muraki

**Affiliations:** Department of Chemistry, Keio University, Yokohama, Japan

**Keywords:** superoxide dismutase, disulfide, crystal structure, dimerization, gram-positive bacteria

## Abstract

Cu/Zn-superoxide dismutase (CuZnSOD) is an enzyme that binds a copper and zinc ion and also forms an intramolecular disulfide bond. Together with the copper ion as the active site, the disulfide bond is completely conserved among these proteins; indeed, the disulfide bond plays critical roles in maintaining the catalytically competent conformation of CuZnSOD. Here, we found that a CuZnSOD protein in *Paenibacillus lautus* (PaSOD) has no Cys residue but exhibits a significant level of enzyme activity. The crystal structure of PaSOD revealed hydrophobic and hydrogen-bonding interactions in substitution for the disulfide bond of the other CuZnSOD proteins. Also notably, we determined that PaSOD forms a homodimer through an additional domain with a novel fold at the N terminus. While the advantages of lacking Cys residues and adopting a novel dimer configuration remain obscure, PaSOD does not require a disulfide-introducing/correcting system for maturation and could also avoid misfolding caused by aberrant thiol oxidations under an oxidative environment.

Most aerobes ranging from prokaryotes to eukaryotes are equipped with Cu/Zn-superoxide dismutase (CuZnSOD), which facilitates the disproportionation of superoxide into hydrogen peroxide and molecular oxygen (1). Removal of the CuZnSOD activity is known to result in deleterious phenotypes such as the suppression of aerobic growth (in budding yeast) (2) and the progressive motor deficits (in mouse and human) (3, 4, 5). Besides, pathogenic bacteria are known to weaken their virulence by losing prophage-encoded CuZnSOD, which is required to cope with the respiratory burst from host cells (6). The activity of CuZnSOD is hence relevant in a variety of physiological processes under aerobic environment.

In order to become an active enzyme, CuZnSOD primarily needs to bind a copper ion at the copper-binding site (Fig. 1) because it functions as the active center for the disproportionation of superoxide. Most of the CuZnSOD proteins also bind a zinc ion at the zinc-binding site (Fig. 1), by which the activity becomes less influenced by the solution pH (7). It is also important to note that CuZnSOD forms an intramolecular disulfide (S–S) bond (Fig. 1); the activity is decreased by breaking the S-S bond through either the reduction or the amino acid substitutions at the Cys residues (8, 9). While CuZnSOD in some species lack the zinc-binding site (10), the S–S bond is completely conserved in CuZnSOD proteins, suggesting an essential and/or regulatory role of the S–S bond in the function of CuZnSOD.

**Figure 1:**
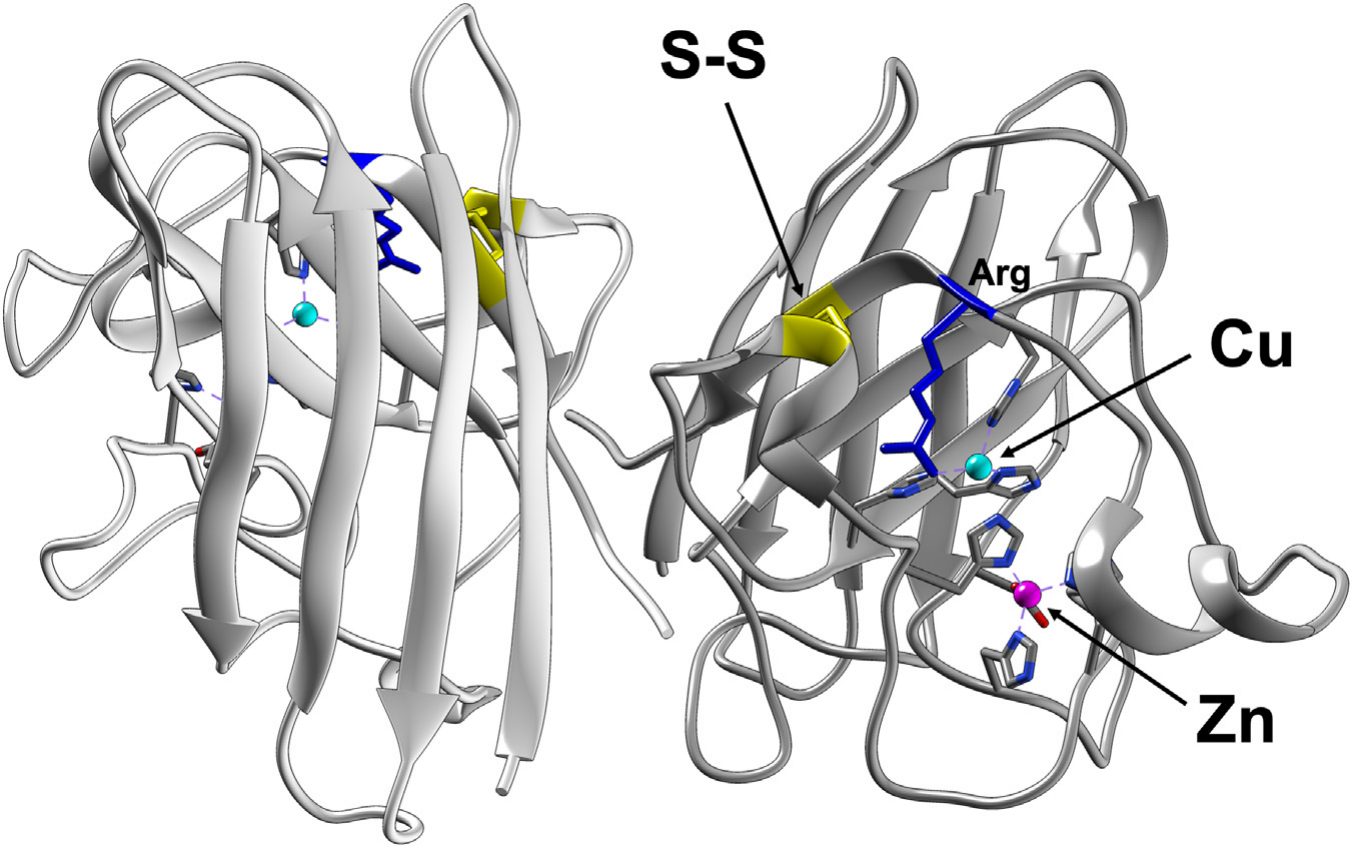
**A three-dimensional structure of human CuZnSOD.** A homodimer of human CuZnSOD (Protein Data Bank ID: 1HL5) is shown with a copper (*cyan*) and zinc (*magenta*) ion and a conserved disulfide bond (*yellow*). The Arg residue (colored *blue*) at the entry site of superoxide to the copper site is also shown. CuZnSOD, Cu/Zn-superoxide dismutase.

A structural significance of the S–S bond in CuZnSOD is to tether a loop to the protein core and thereby shape an entry site of the substrate O_2_^−^ leading to the catalytic copper ion. The S–S bond is also proposed to have influence on the configuration of a conserved Arg at the entry site (Fig. 1), the positive charge of which appears to play important roles in attracting O_2_^−^ and electrostatically steering it to the copper ion (11, 12). In general, an S–S bond is unstable in reducing environment of the cytosol where eukaryotic CuZnSOD localizes (13). Nonetheless, eukaryotic CuZnSOD forms a tight homodimer so as to occlude the S–S bond of each subunit at the dimer interface (Fig. 1); thereby, the S–S bond in CuZnSOD is protected from intracellular reducing agents such as glutathione and hence highly resistant to the reduction (14). Prokaryotic CuZnSOD is also equipped with the intramolecular S–S bond formed in eukaryotic CuZnSOD but exists as either a monomer or an alternative homodimer, in which the S–S bond is exposed to the solvent. This is consistent with the fact that CuZnSOD from Gram-negative bacteria is localized in oxidizing environment of the periplasmic space (15). Together with the copper ion as the active site, therefore, the intramolecular S–S bond has been considered to play a primary role in maintaining the catalytically competent conformation and expressing the CuZnSOD activity.

To check if the S–S bond is indeed conserved in CuZnSOD, we searched a “Protein Family Models” database provided by the National Center for Biotechnology Information (NCBI) and found that the proteins registered as “Cu_amine_oxidN1 and Cu^_^Zn_superoxide dismutase domain–containing protein” have no Cys residues in their primary sequences and thus lack the S–S bond. According to the database, furthermore, the Cys-less CuZnSOD is a unique protein found in the *Paenibacillaceae*, a family of Gram-positive bacteria. Nonetheless, it remains unknown whether this uncharacterized protein can function as CuZnSOD without the conserved S–S bond.

Here, we show that the Cys-less CuZnSOD from *Paenibacillus lautus* (PaSOD) exhibits a significant level of the superoxide-dismutation activity. We performed structural and biochemical characterizations of PaSOD and confirmed that the C-terminal CuZnSOD domain of PaSOD had the activity albeit with no S–S bond. Furthermore, PaSOD was found to form a homodimer through the N-terminal domain with a novel protein fold. Taken together, we propose that CuZnSOD can exert the enzymatic activity even without the conserved S–S bond and further discuss a possible advantage to lacking the Cys residues in PaSOD under oxidative environment in particular.

## Results

### PaSOD has no Cys residue in its primary sequence

As described above, we found that CuZnSOD proteins registered as “Cu_amine_oxidN1 and Cu-Zn_superoxide dismutase domain–containing protein (Arch. ID: 10936698)” in the NCBI database have no Cys residue. The NCBI reference sequence database (RefSeq) of those Cys-less CuZnSOD proteins (PaSOD) is comprised of 17 highly conserved sequences from *Aneurinibacillus*, *Cohnella*, *Paenibacillus*, and *Saccharibacillus*, all of which are in a family of *Paenibacillaceae*, Gram-positive bacteria (Fig. S1). We also performed a BLAST search in UniProt using UniRef100 as a target database and identified PaSOD proteins in several species of *Paenibacillus*, *Cohnella*, *Saccharibacillus*, *Aneurinbacillus*, and *Ammoniphilus*, all of which belong to the family *Paenibacillaceae*. Only one apparent exception that has PaSOD is *Geobacillus* sp. (strain Y412MC10), which belonged to the family *Bacillaceae*; nonetheless, this species is now considered as a strain of *P. lautus* (*Paenibacillus* sp. Y412MC10) (16).

In order to characterize PaSOD as a novel Cys-free CuZnSOD, we obtained *P. lautus* NBRC 15380 from the Biological Resource Center, NITE (NBRC); according to the database in the NCBI, two SOD family proteins (*i.e.*, PaSOD) are annotated in the genome of *P. lautus* NBRC 15380: WP_246059278.1 and WP_246059286.1 (Fig. S2). Fig. S2 also shows that the N-terminal approximately 30 amino acids of PaSOD are predicted to be a signal peptide transported by the Sec translocon and cleaved by signal peptidase I (SignalP-6.0 provided by DTU Health Tech). In this study, therefore, WP_246059278.1 and WP_246059286.1 without the predicted signal peptide was called as PaSOD-1 and PaSOD-2, respectively.

**Figure 2:**
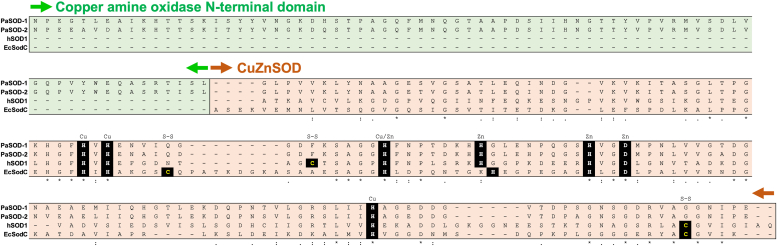
**Amino acid sequence of PaSOD-1 and -2 from *Paenibacillus lautus.*** The amino acid sequence of PaSOD-1 and -2 (without the N-terminal signal sequence) from *P. lautus* is aligned with that of hSOD1 and EcSodC, which was performed by ClustalW. The regions regarded as “copper amine oxidase N-terminal domain” and “superoxide dismutase [CuZnSOD] domain” are also indicated. The amino acid residues responsible for binding of a copper and zinc ion as well as the disulfide-bonding Cys residues are emphasized as indicated. EcSodC, *Escherichia coli* CuZnSOD; hSOD1, human CuZnSOD; PaSOD, Cys-less CuZnSOD protein in *Paenibacillus lautus.*

As shown in Figure 2, PaSOD-1 and -2 from *P. lautus* NBRC 15380 have almost the same amino acid sequence (93% identity) and are comprised of the domain similar to the copper amine oxidase (CAO) N-terminal domain (Asn50–Leu124) followed by the CuZnSOD domain (Gly125–Glu269). The CuZnSOD domain of PaSOD-1 and -2 shows slightly higher sequence identity with human CuZnSOD (hSOD1, *ca*. 32%) compared with that with *Escherichia coli* CuZnSOD (EcSodC, *ca*. 26%). All seven amino acid residues binding a copper and zinc ion are conserved, but the two Cys residues required for the conserved intramolecular S–S bond are absent and replaced with Phe and Gly in PaSOD-1 and -2.

### Recombinant PaSOD-1 and -2 exhibit the activity of SOD

To test if PaSOD-1 and -2 function as authentic CuZnSOD even in the absence of the conserved S–S bond, PaSOD-1 and -2 without the signal peptide were expressed in *E. coli* and purified with Ni^2+^-affinity chromatography followed by gel-filtration chromatography (see the Experimental procedures section). As-purified PaSOD-1 and -2 were found as a metal-deficient apo form that contained almost no copper but a nominal amount of zinc (<10%). When expressed in *E. coli* cultured with the media containing 2 mM CuSO_4_ and 30 μM ZnSO_4_, nonetheless, PaSOD-1 and -2 were obtained as partially metallated forms containing copper (∼50%) and zinc (∼30%). Furthermore, purified PaSOD-1 and -2 were fully metallated *in vitro* with an equimolar amount of copper and zinc by addition of 1.2-fold excess amounts of CuSO_4_ and ZnSO_4_ followed by the removal of unbound metal ions with ultrafiltration. Therefore, PaSOD-1 and -2 were shown to tightly bind a copper and zinc ion, which is consistent with the fact that the copper- and zinc-binding sites are conserved in the CuZnSOD domain of PaSOD-1 and -2.

**Figure 3:**
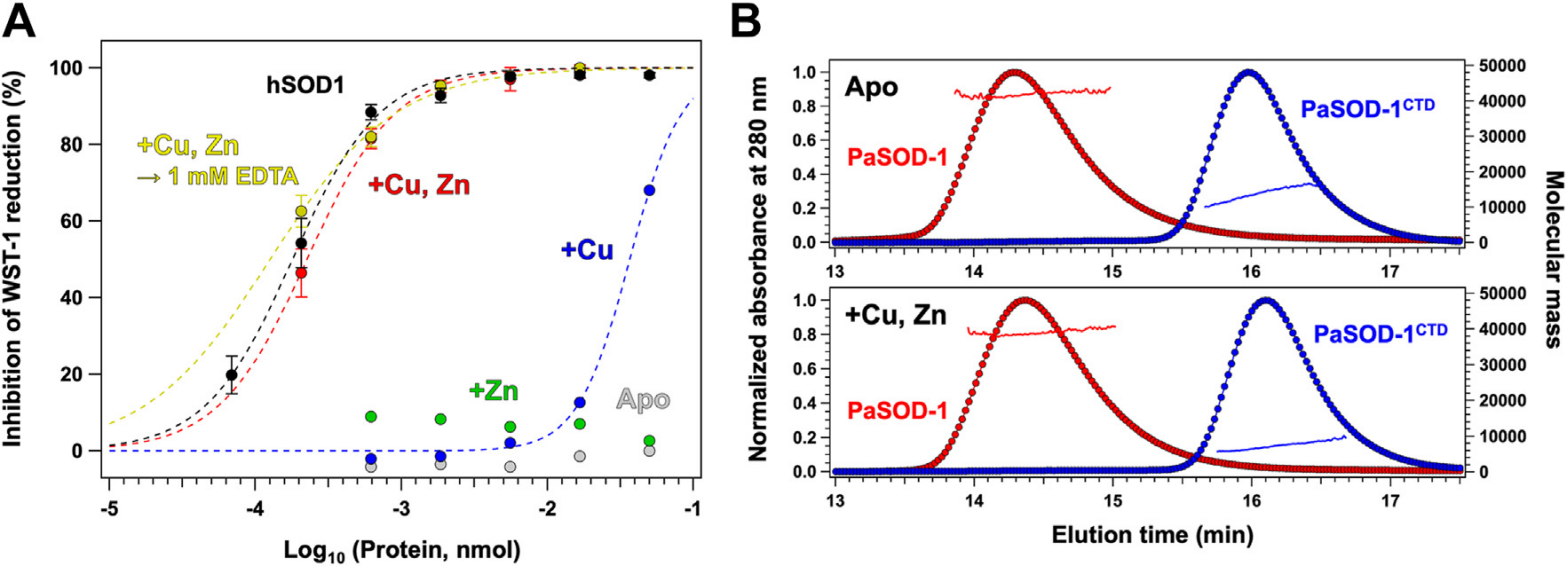
**PaSOD-1 exhibits enzymatic activity as CuZnSOD.***A*, activity assay was performed using (*gray*) as-isolated apo form of PaSOD-1, PaSOD-1 with an equimolar amount of either (*green*) Zn^2+^ or (*blue*) Cu^2+^, and (*red*) PaSOD-1 with an equimolar amount of both Cu^2+^ and Zn^2+^. Following addition of an equimolar amount of both Cu^2+^ and Zn^2+^, PaSOD-1 was further treated with 1 mM EDTA, and the activity assay was performed (*yellow*). As a positive control, the activity of human CuZnSOD in the holo form was also assayed (*black*). *B*, size-exclusion chromatograms of PaSOD-1 proteins (20 μM in the MN buffer), which were monitored at 280 nm and normalized for comparison (*left axis*), were shown with the molecular mass estimated by MALS (*right axis*): the *upper panel*, PaSOD-1 (*red*) and PaSOD-1^CTD^ (*blue*) in the apo form: the *lower panel*, PaSOD-1 (*red*) and PaSOD-1^CTD^ (*blue*) in the holo form. CuZnSOD, Cu/Zn-superoxide dismutase; MALS, multiangle light scattering; PaSOD-1, Cys-less CuZnSOD protein in *Paenibacillus lautus*; PaSOD-1^CTD^, C-terminal domain of PaSOD-1.

We first examined the CuZnSOD activity of PaSOD-1, which can be represented by the amount of the protein that gives 50% inhibition of the WST-1 reduction by superoxide (IC_50_) (17). In the presence of equimolar copper and zinc ions, PaSOD-1 inhibited the WST-1 reduction and exhibited a significant level of activity (IC_50_ = 0.20 ± 0.04 pmol), which was comparable to that of hSOD1 (IC_50_ = 0.18 ± 0.02 pmol) (Fig. 3*A*, *red* and *black*). This is also comparable to the activity of EcSodC (IC_50_ = 5.4 ng = 0.34 pmol) that we previously reported (18). The activity is thus considered to be similar among eukaryotic, prokaryotic CuZnSOD, and PaSOD, which is indeed consistent with the fact that various types of SODs including Mn-, Fe-, and Ni-containing SODs exhibit similar levels of the activity because of the rates of reaction with superoxide near the diffusion-controlled limit (19). The activity of PaSOD-1 was not affected by addition of 1 mM EDTA, a strong divalent metal chelator (Fig. 3*A*, *red* and *yellow*), suggesting tight binding of a copper and zinc ion in PaSOD-1. No inhibition of WST-1 was observed with PaSOD-1 in the absence of any metals (apo) or in the presence of Zn^2+^ but not Cu^2+^ (Fig. 3*A*, *gray* and *green*). Addition of Cu^2+^ alone to apo-PaSOD-1 resulted in an almost negligible but detectable level of the activity (Fig. 3*A*, *blue*); this would be due to the activation of a small amount of the zinc-bound form that was contaminated in the apo-PaSOD-1 sample. The CuZnSOD activity of PaSOD-2 was also examined, and almost the same results with those of PaSOD-1 were obtained (Fig. S3*A*). Therefore, PaSOD-1 and -2 can function as CuZnSOD by binding a copper and zinc ion plausibly at the respective binding sites in the CuZnSOD domain: indeed, we confirmed a significant level of the activity in the isolated CuZnSOD domain of PaSOD-1 and -2 (PaSOD-1^CTD^ and PaSOD-2^CTD^, respectively; Fig. 2) by adding equimolar copper and zinc ions (IC_50_: 0.33 ± 0.04 pmol in PaSOD-1^CTD^ and 0.11 ± 0.01 pmol in PaSOD-2^CTD^).

### Roles of NTD of PaSOD-1 and -2 in homodimerization

The CuZnSOD activity of PaSOD-1/2 was not affected by the deletion of their N-teminal domain (PaSOD-1^NTD^ and PaSOD-2^NTD^). According to the annotation by Pfam (PF07833), this domain originates from the N-terminal domain of CAO and can interact with each other, leading us to suspect that PaSOD-1/2^NTD^ plays a role in the homodimerization of PaSOD-1/2. We thus analyzed the quaternary structures of PaSOD-1/2 and PaSOD-1/2^CTD^ with the size-exclusion chromatography followed by the multiangle light scattering (SEC–MALS). Both PaSOD-1 and -2 in the apo state were found to be eluted as a single peak with a molar mass of 45,000 that was estimated by MALS (Fig. 3*B* for PaSOD-1 and Fig. S3*B* for PaSOD-2). The calculated molar mass values of PaSOD-1 and -2 based upon their primary sequences are 23,242 and 23,278, respectively; therefore, apo-PaSOD-1 and -2 were considered to form a homodimer in solution. In contrast, both apo-PaSOD-1/2^CTD^ were eluted as a single peak with an estimated molar mass of 13,300, which closely matches the calculated molar mass (15,082 for PaSOD-1^CTD^ and 15,098 for PaSOD-2^CTD^) (Figs. 3*B* and S3*B*). Furthermore, binding of the copper and zinc ion did not affect the quaternary structure of PaSOD-1/2 (homodimer) and PaSOD-1/2^CTD^ (monomer) (Figs. 3*B* and S3*B*), which is in sharp contrast to the homodimerization of human and yeast CuZnSOD upon binding of the metal ions (8, 20). Accordingly, PaSOD-1/2^CTD^ is expected to exist as a monomer, and PaSOD-1/2^NTD^ plays a critical role in the homodimerization of PaSOD-1/2.

### PaSOD-1^NTD^ is characterized by unprecedented fold and function

To examine roles of the homodimerization through the N-terminal domain of PaSOD, the thermal stability was first compared between full-length PaSOD-1 and PaSOD-1^CTD^ variant by differential scanning fluorometry. PaSOD-1^CTD^ with equimolar Cu^2+^ and Zn^2+^ exhibited the melting temperature (*T*_m_) at 38.0 °C, whereas *T*_m_ of the full-length PaSOD-1 protein with equimolar Cu^2+^ and Zn^2+^ was found to be 50.5 °C. Therefore, the N-terminal domain of PaSOD is considered to stabilize the full-length protein.

**Figure 4:**
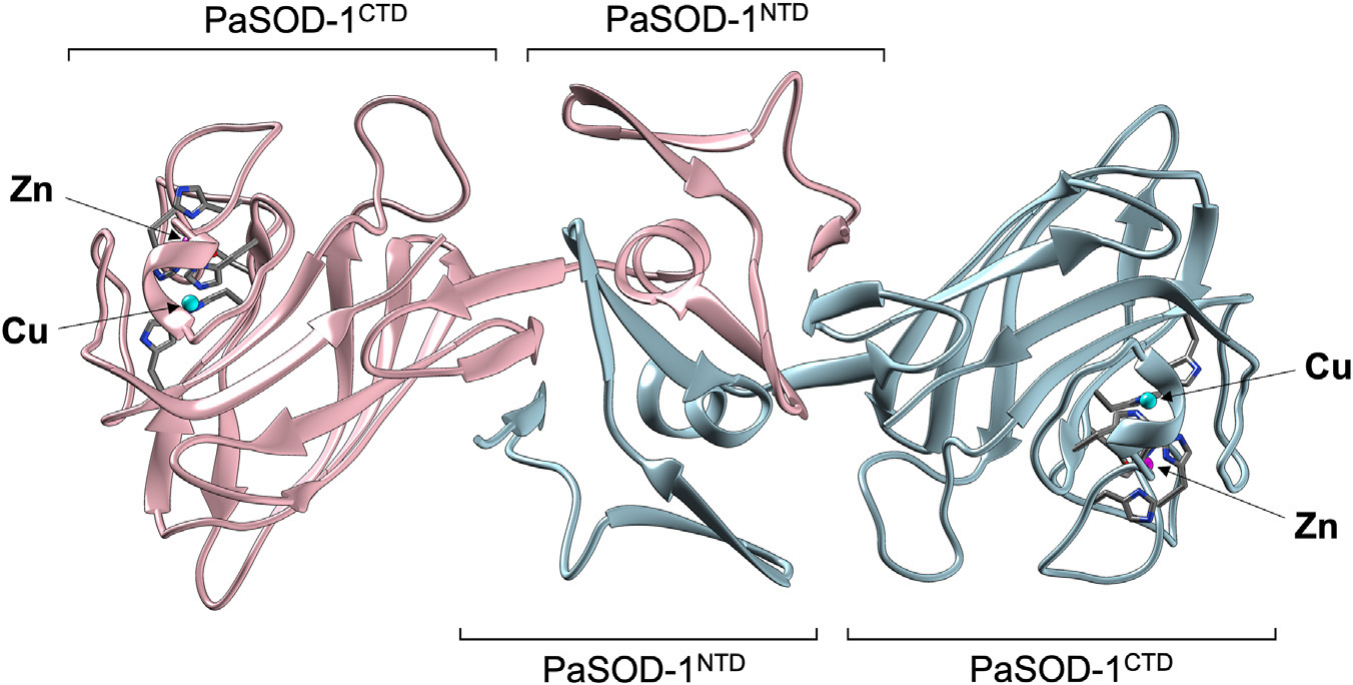
**PaSOD-1 forms a homodimer in a novel configuration.** The overall structure of PaSOD-1 is revealed by X-ray crystal structural analysis of the dataset collected at the wavelength of 1.275 Å and shown in a *ribbon model* using a software UCSF Chimera (Protein Data Bank ID: 8IMD). The N-terminal domain indicated as PaSOD-1^NTD^ is responsible for the homodimerization of PaSOD-1. PaSOD-1^CTD^ exhibits an immunoglobulin-fold typical of CuZnSOD (Fig. 1) and binds a copper (*cyan*) and zinc (*magenta*) ion. The ligands for binding those metal ions are shown in a *stick model*. PaSOD, Cys-less CuZnSOD protein in *Paenibacillus lautus*.

To reveal a three-dimensional structure of PaSOD-1/2, we next performed X-ray structural analysis on PaSOD-1/2 supplemented with a copper and zinc ion. We obtained crystals of both PaSOD-1 and PaSOD-2 but failed to complete structure modeling and refinement of PaSOD-2 because of a poor electron density map (data not shown, see the Experimental procedures section). In contrast, we successfully solved the crystal structures of PaSOD-1 by the single-wavelength anomalous diffraction method using wavelengths of Cu and Zn absorption edges at 1.50 Å and 1.45 Å resolution, respectively (Fig. 4 and Table S1). We used the structure from data collected at a wavelength of Zn absorption edge (1.275 Å) to discuss the PaSOD-1 structure unless otherwise noted. A crystal asymmetric unit contains two molecules of PaSOD-1, each of which consists of two distinct domains: namely, PaSOD-1^NTD^ and PaSOD-1^CTD^ (Fig. 4). The crystal structure of PaSOD-2 could not be solved experimentally; however, 207 of the total 220 amino acid residues are identical between PaSOD-1 and -2, and the relevant residues including the metal-binding sites are conserved. PaSOD-2 is thus well expected to have essentially the same structure with that of PaSOD-1. Consistent with the SEC–MALS analysis, furthermore, PaSOD-1^NTD^ but not PaSOD-1^CTD^ is confirmed to be responsible for the homodimerization of PaSOD-1. This is a novel configuration of the subunits in dimeric CuZnSOD proteins.

**Figure 5:**
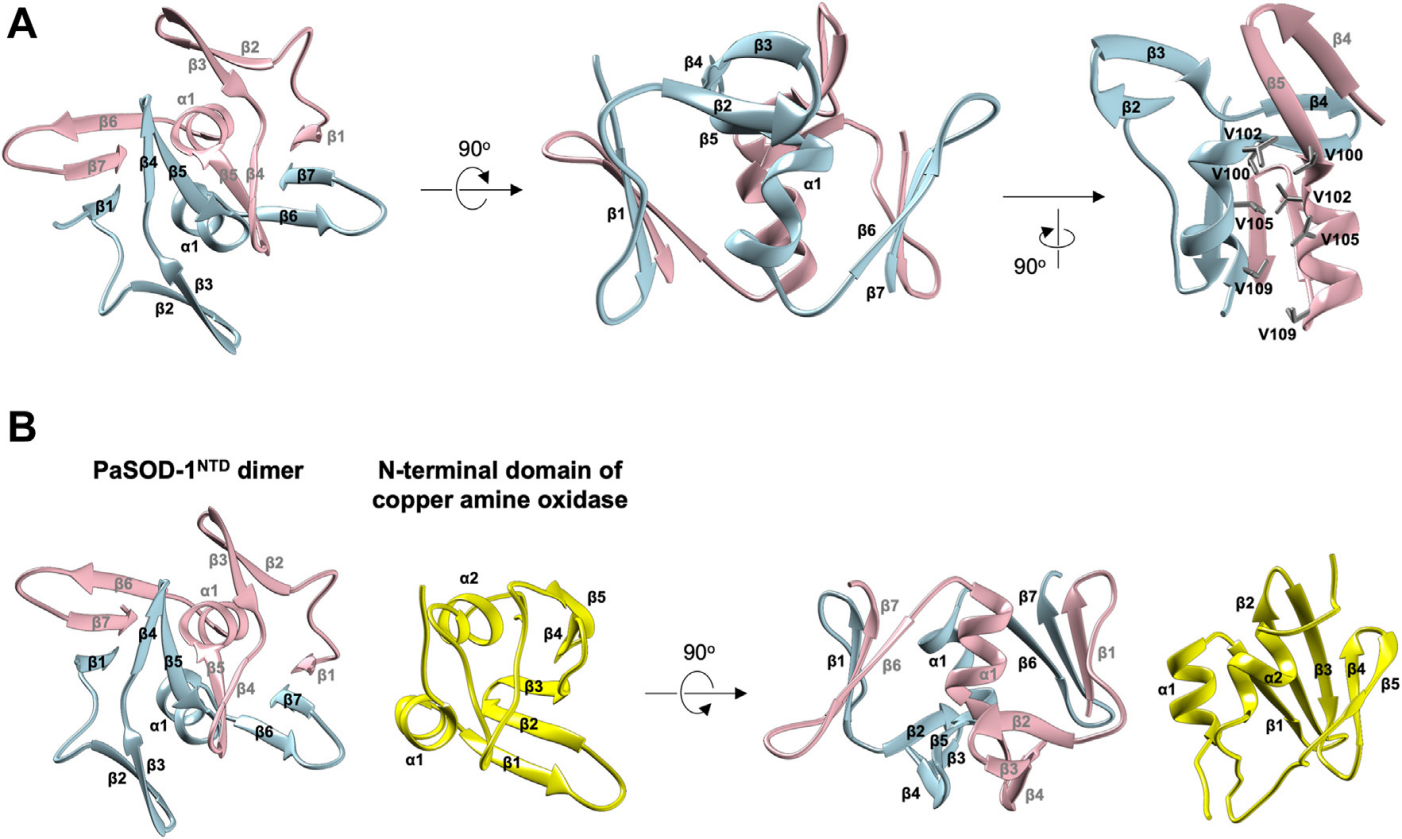
**A novel fold of PaSOD-1**^**NTD**^**plays a role in the homodimerization of PaSOD-1.***A*, the structure of dimeric PaSOD-1^NTD^ (*pink* and *sky blue*) in a *ribbon model* is extracted from the overall structure (Fig. 4) and rotated vertically and horizontally by 90° as indicated. β-strands and α-helix in PaSOD-1^NTD^ are designated as β1–β7 and α1, respectively, and valine residues forming hydrophobic clusters are shown in a *stick model*. *B*, structural comparisons between dimeric PaSOD-1^NTD^ and the N-terminal domain of *Escherichia coli* copper amine oxidase (Protein Data Bank ID: 1OAC) are performed by their alignment using Matchmaker in UCSF Chimera. PaSOD, Cys-less CuZnSOD protein in *Paenibacillus lautus.*

To get more structural insights into this unique configuration of PaSOD-1 subunits, we first highlighted a region of PaSOD-1^NTD^ in Figure 5*A*. PaSOD-1^NTD^ is composed of seven β-strands and one α-helix; we searched for proteins with similar structures to that of PaSOD-1^NTD^ by using the Dali server but found no results. Upon interaction between PaSOD-1^NTD^ domains, β1 and β7 of PaSOD-1^NTD^ are found to form parallel β-sheets with β7 and β1 of the other PaSOD-1^NTD^, respectively (Fig. 5*A*, *left*). The β-sheets are further extended through intrasubunit antiparallel interaction between β6 and β7. Also, in the dimeric configuration, another antiparallel β-sheet was observed between β5 strands, which appears to form an incomplete β-barrel through the antiparallel interaction between β4 and β5 (Fig. 5*A*, *left*). Furthermore, the α-helices from each of the subunits are held together by a hydrophobic cluster made of valine residues (V100, V102, V105, and V109) and thereby form an intersubunit two-helix bundle (Fig. 5*A*, *right*). Therefore, PaSOD-1^NTD^ functions as a novel dimerization domain by forming β-sheets and a helix bundle between the subunits.

Based upon the primary sequence analysis, PaSOD-1^NTD^ has been categorized in the family of the N-terminal domain of CAO (Pfam ID: PF07833). We thus compared the structure of PaSOD-1^NTD^ with that of the N-teminal domain (residue numbers: 33–117) of *E. coli* CAO (CAO-N), but they appear not to be structurally similar with each other (Fig. 5*B*). More precisely, the α1–β6–β7 region in PaSOD-1^NTD^ (*blue* in Fig. 5*B*) was found to be three-dimensionally well aligned with the α1–β–β2 region of CAO-N (*yellow* in Fig. 5*B*), but the region from β1 to β4 in PaSOD-1^NTD^ is missing in CAO-N. Besides, three β-strands (β3, β4, and β5) and one α-helix (α2) in CAO-N are missing in PaSOD-1^NTD^. Nonetheless, when CAO-N is compared with the dimeric state of PaSOD-1^NTD^, the β3 strand of CAO-N is aligned with β1 of the other PaSOD-1^NTD^ molecule (*pink* in Fig. 5*B*) albeit with the opposite direction. Moreover, the α2 helix of CAO-N corresponds to α1 of the other PaSOD-1^NTD^ molecule (*pink* in Fig. 5*B*). As observed in the intermolecular interaction through the α1 helices in the PaSOD-1^NTD^ dimer (Fig. 5*A*, *right*), the interaction between α1 and α2 within CAO-N is also mainly hydrophobic. Taken together, PaSOD-1^NTD^ is partially similar to the protein family of the N-terminal domain of CAO but is considered to be a novel fold.

### PaSOD-1^CTD^ possesses a typical fold of CuZnSOD but has no S–S bond

**Figure 6:**
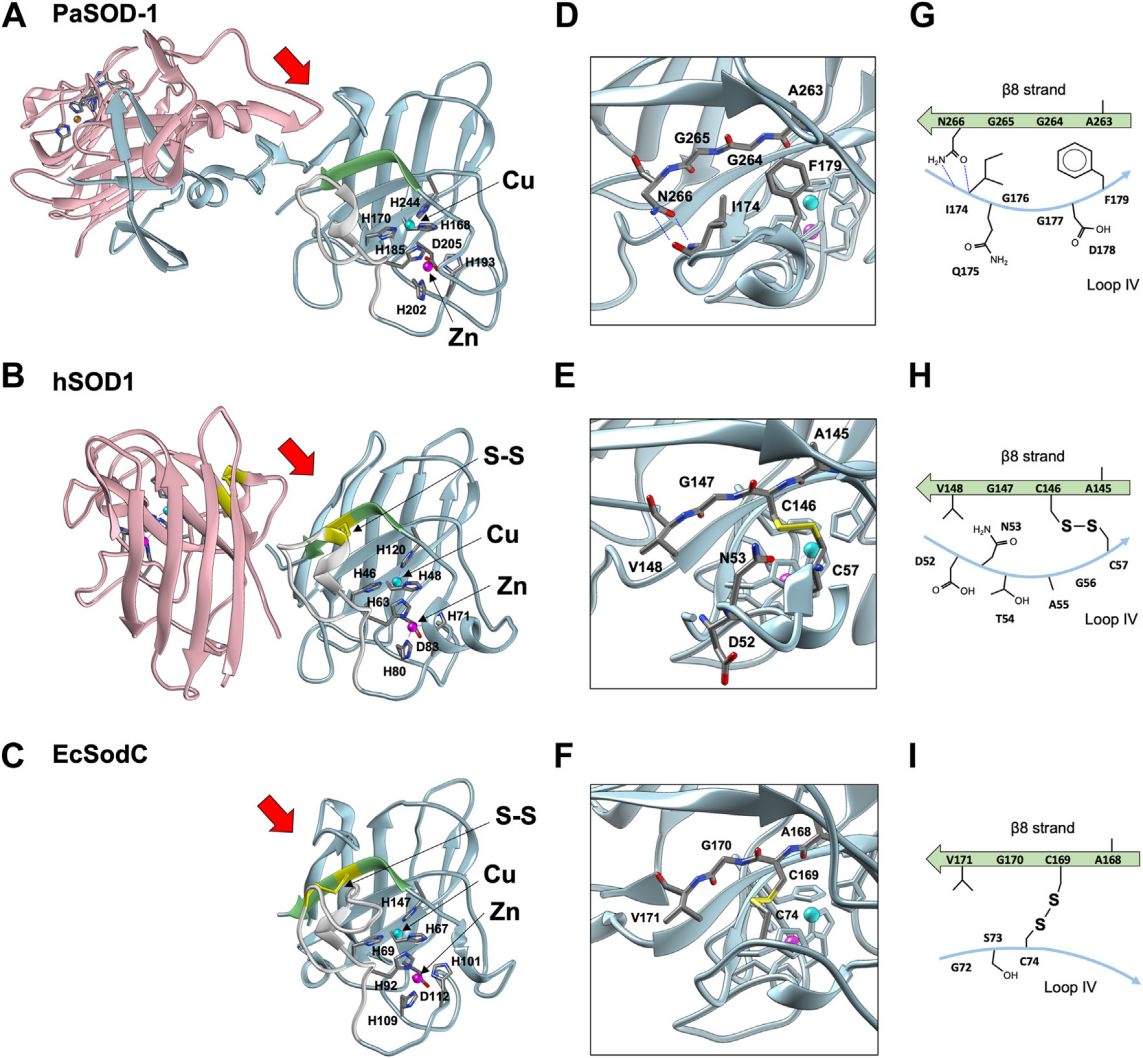
**Hydrophobic interactions replace the conserved disulfide bond in PaSOD-1.***A*–*C*, the overall structures of (*A*) PaSOD-1 dimer, (*B*) hSOD1 (Protein Data Bank ID: 1HL5), and (*C*) EcSodC (Protein Data Bank ID: 1ESO) are shown in a *ribbon model*. The PaSOD-1 dimer is shown by aligning one of PaSOD-1^CTD^ (colored in *sky blue*) with the subunit of hSOD1 and EcSodC (also colored in *sky blue*). The conserved disulfide bond in hSOD1 and EcSodC is colored *yellow*, and the amino acid residues ligating a copper (*cyan*) and zinc (*magenta*) ion are shown in a *stick model*. β8 strand and loop IV in a subunit of PaSOD-1, hSOD1, and EcSodC are colored in *green* and *white*, respectively. *D*–*F*, the magnified images of the subunits of (*D*) PaSOD-1, (*E*) hSOD1, and (*F*) EcSodC, which are looked from the direction shown as *red arrows* in (*A*–*C*), are shown. *G*–*I*, the amino acid configurations between β8 strand and loop IV are schematically represented in (*G*) PaSOD-1, (*H*) hSOD1, and (*I*) EcSodC. EcSodC, *Escherichia coli* CuZnSOD; hSOD1, human CuZnSOD; PaSOD, Cys-less CuZnSOD protein in *Paenibacillus lautus*.

We next focus on structural features of the other domain, PaSOD-1^CTD^ (Fig. 4). PaSOD-1^CTD^ assumes an immunoglobulin-like fold commonly observed for CuZnSOD, which is mainly composed of eight β-strands. This is supported by the fact that superposition of the α-carbon backbone atoms of PaSOD-1^CTD^ with those of hSOD1 (Protein Data Bank ID: 1HL5) and EcSodC (Protein Data Bank ID: 1ESO) gave an rms difference of 0.663 Å (Fig. 6, *A* and *B*) and 1.206 Å (Fig. 6, *A* and *C*) by using PyMOL (Schrödinger, LLC), respectively. PaSOD-1^CTD^ was found to bind a copper and zinc ion, the positions of which were determined based upon the anomalous scattering data collected at wavelengths 1.373 Å (Cu signal) and 1.275 Å (Cu and Zn signals) (Fig. S4 and Table S1). Their binding sites are almost overlapped with those in hSOD1 and EcSodC; a copper ion in PaSOD-1^CTD^ is ligated through His168, His170, His185, and His244, whereas a zinc ion through His185, His193, His202, and Asp205 (Fig. 6*A*). His185 is a so-called bridging ligand binding both a copper and zinc ion. Those metal-binding features are consistent with our expectation based upon the primary sequence analysis (Fig. 2).

As indicated in hSOD1 (Fig. 6*B*) and EcSodC (Fig. 6*C*), the intramolecular S–S bond (colored by *yellow*) is conserved in all CuZnSODs and has a role in connecting the loop (loop IV, colored by *light gray*) with the β-barrel core through the β8 strand (colored by *green*). The conformation of loop IV in PaSOD-1 is similar to that of hSOD1 but distinct from that of EcSodC, which is larger in length than that of PaSOD-1 and hSOD1 (Fig. 6, *A*–*C*). Reduction of the S–S bond is known to significantly decrease the conformational stability of both hSOD1 (21) and EcSodC (22); therefore, we wondered how PaSOD-1 lacking the S–S bond maintains the interaction between loop IV and the β8 strand. When we looked on loop IV–β8 strand interaction from the direction pointed by a *red arrow* in Figure 6, *A* and *B*, the S–S bond in hSOD1 was found to be replaced with a phenyl group of Phe179 and a hydrogen of Gly264 in PaSOD-1 (Fig. 6, *D* and *E*). Moreover, Asp52 in loop IV of hSOD1 points outward (Fig. 6*E*) but is replaced with Ile174 pointing inward in PaSOD-1 (Fig. 6*D*), where a hydrophobic cluster (Ile174, Gly176, Gly177, Phe179, Gly264, and Gly265) is formed between loop IV and β8 strand (Fig. 6*G*). In contrast, in hSOD1, the hydrophilic residue of Asn53 fills in the space surrounded by loop IV and β8 strand (Fig. 6, *E* and *H*). Another notable feature in PaSOD-1 is that the side chain carbonyl and amide groups of Asn266 form hydrogen bonds with the main chain amide and carbonyl group of Ile174, respectively (Fig. 6, *D* and *G*), whereas these hydrogen bonding interactions are missing in hSOD1 (Fig. 6, *E* and *H*). While the relative configuration of loop IV and β8 strand in EcSodC is not well matched with that of PaSOD-1 and hSOD1 (Fig. 6, *A*–*C*), the S–S bond appears to play a primary role in the loop IV/β8 strand interaction, and the hydrophobic cluster as well as the hydrogen-bonding interactions observed in PaSOD-1 are absent in EcSodC (Fig. 6, *F* and *I*). In PaSOD-1, therefore, loop IV is connected to the β8 strand mainly through the hydrophobic interactions together with the hydrogen bonds, which replaces the disulfide bonding interaction in hSOD1 and EcSodC.

**Figure 7:**
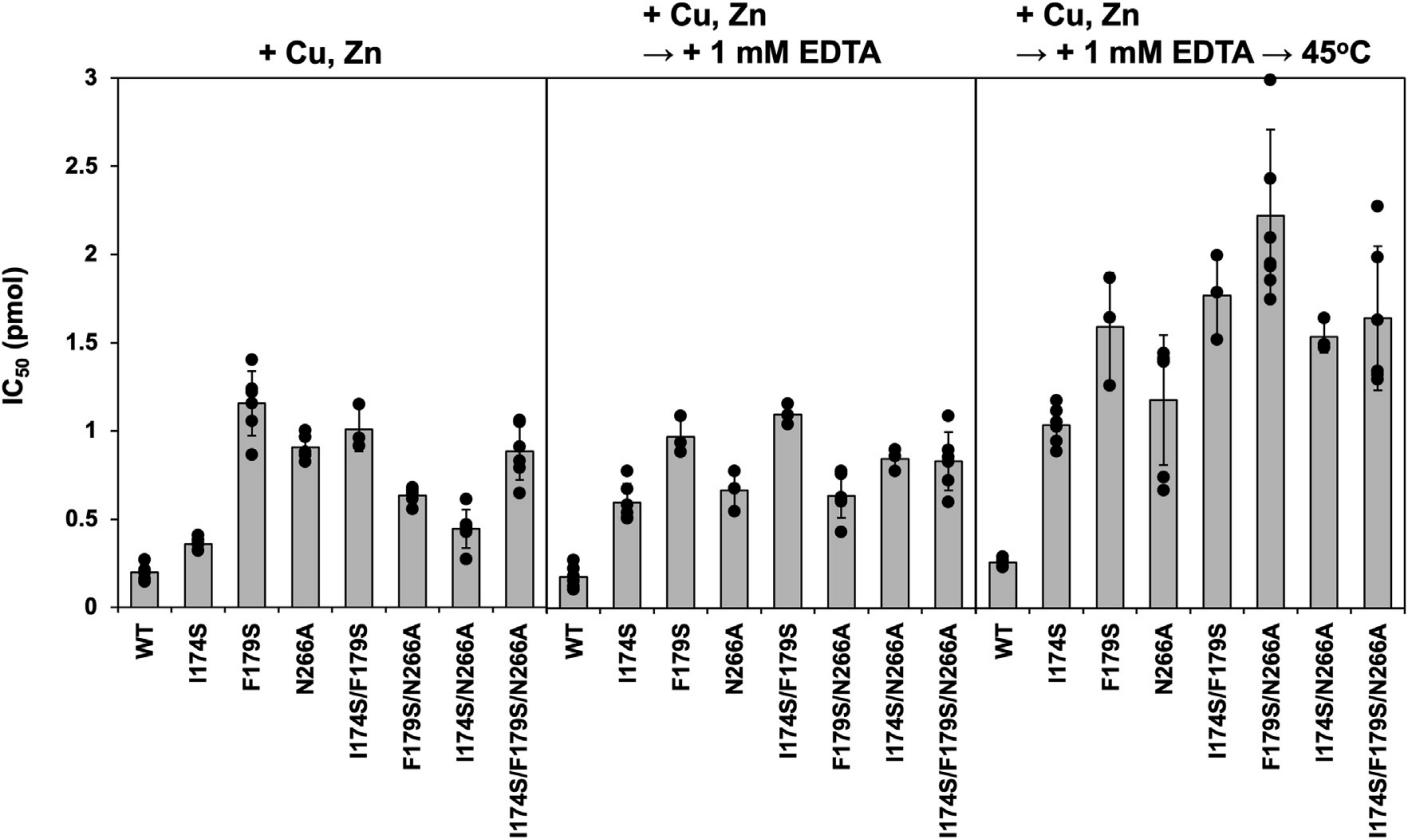
**Disrupting the interaction between β8 and loop IV leads to decreased activity of PaSOD-1.** PaSOD-1 and the proteins with indicated mutations containing an equimolar amount of a Cu^2+^ and Zn^2+^ ion (*left panel*) were examined with the CuZnSOD activity assay, and the activities were shown as the amount of the protein (IC_50_) that gives 50% inhibition of the WST-1 reduction by superoxide. Besides, PaSOD-1 proteins containing Cu^2+^ and Zn^2+^ ions were incubated with 1 mM EDTA at 25 °C (*middle panel*) or 45 °C (*right panel*) for an hour, and the activity assay was then performed. Averages were shown as bars with individual data points and error bars (standard deviation). PaSOD, Cys-less CuZnSOD protein in *Paenibacillus lautus*.

To test if the hydrophobic interaction plays roles in the function of PaSOD-1, the activity assay was performed using mutant PaSOD-1 with substitution of Ser for Ile174/Phe179. Besides, substitution of Ala for Asn266 was examined to evaluate significance of the hydrogen-bonding interaction in the maintenance of the enzymatically competent conformation of PaSOD-1. As summarized in Figure 7 (*left panel*), the enzymatic activity in the presence of an equimolar amount of copper and zinc ions was decreased (*i.e.*, IC_50_ was increased) in all mutant PaSOD-1 proteins with I174S, F179S, N266A, and their combinations. While addition of 1 mM EDTA at 25 °C did not significantly affect the activity of the PaSOD-1 proteins (*middle panel* in Fig. 7), further incubation at 45 °C with EDTA led to decrease of the activity in the mutant PaSOD-1 proteins (*right panel* in Fig. 7). In contrast, wild-type PaSOD-1 was found to retain high enzymatic activity even after treatment with EDTA at 45 °C (Fig. 7). Collectively, we suggest that hydrophobic and hydrogen-bonding interactions through Ile174, Phe179, and Asn266 maintain the connection between β8 strand and loop IV and thereby stabilize the catalytically competent conformation of PaSOD-1.

### Expression of PaSOD-1/2 in *P. lautus*

While PaSOD-1/2 was expected to be translated as a novel type of a multidomain protein, we just wondered if the protein really exists and functions as the full-length form in *P. lautus*. To confirm the expression of full-length PaSOD-1/2 in *P. lautus* NBRC 15380, therefore, we first attempted to make the *P. lautus* strain in which the PaSOD-1/2 genes were deleted by replacing it with an antibiotic resistance gene. Nonetheless, *P. lautus* NBRC 15380 was resistant to antibiotics and found to grow on 2xYT agar plates supplemented with either kanamycin, streptomycin, or chloramphenicol (data not shown) possibly because of the sporulation ability, making it difficult to delete the PaSOD-1/2 genes. We hence attempted the immunochemical detection of PaSOD-1/2 in *P. lautus* NBRC 15380 by using a polyclonal antibody that was raised in rabbits immunized with the peptide covering Gly246–Gly259 in PaSOD-1.

**Figure 8:**
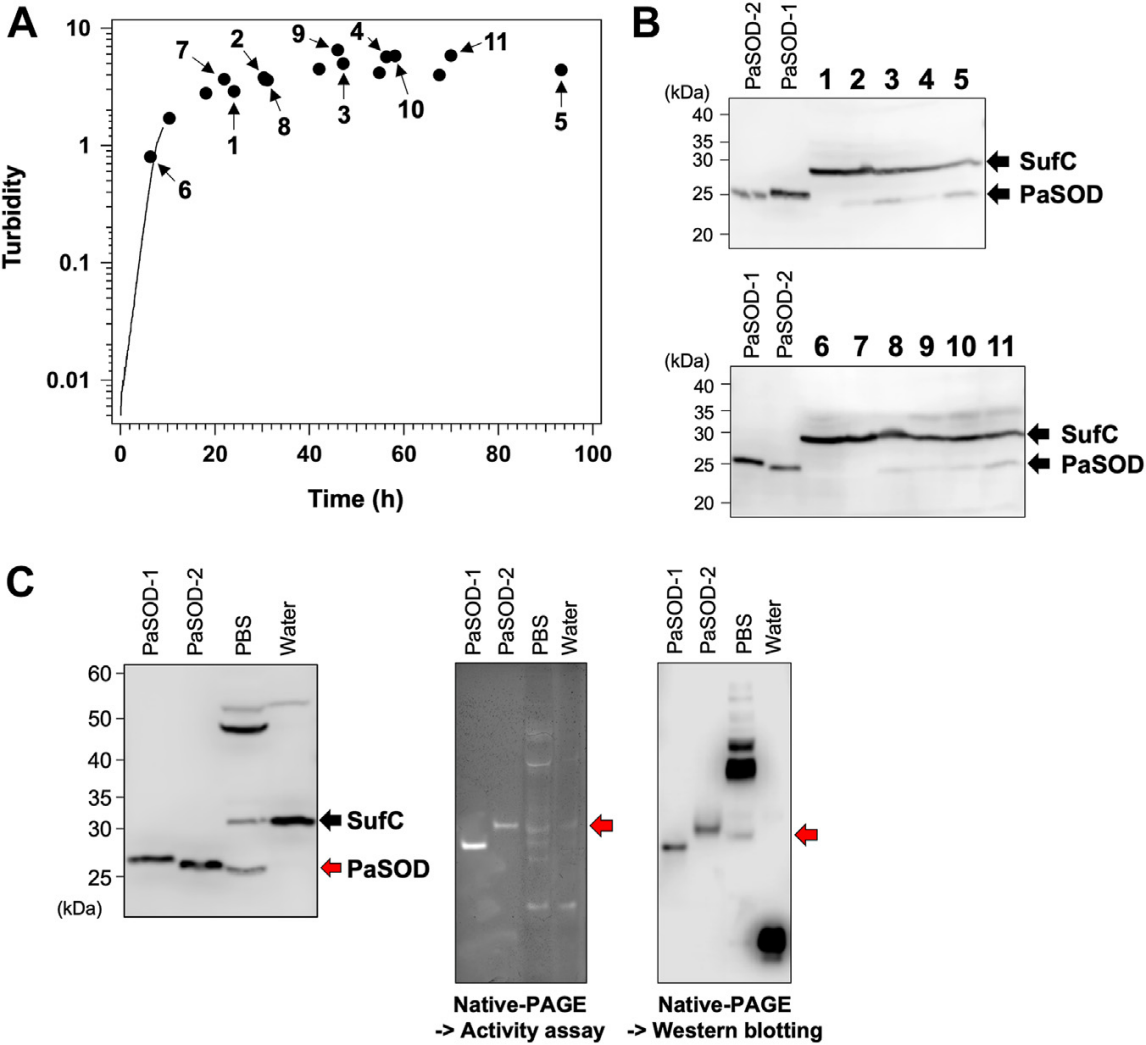
**Expression of endogenous PaSOD in *Paenibacillus lautus* NBRC 15380.***A*, a representative growth curve of *P. lautus* in 2xYT with trace elements is shown. The turbidity was monitored automatically with the rocking incubator (TVS062CA, ADVANTEC: see the Experimental procedures section) and shown as a curve in the first ∼10 h. After ∼10 h of incubation, the turbidity (the absorbance at 660 nm) exceeded 2, an upper limit for the turbidity accurately measured; therefore, the turbidity was manually measured by diluting the *P. lautus* cultures at the indicated time (*circles*). *B*, *P. lautus* was collected at the time indicated in (*A*) (shown as 1–11), lysed, and then analyzed by Western blotting with anti-PaSOD antibody. *C*, the *P. lautus* lysates incubated for 5 days were semipurified as described in the Experimental procedures section; the fractions eluted from the HiTrap Phenyl FF column with PBS and water were analyzed by (*left*) Western blotting, (*middle*) in-gel superoxide dismutase activity assay, and (*right*) immunoblotting followed by native-PAGE. The bands observed in the immunoblots (*left* and *right*) were detected with anti-PaSOD. As controls, recombinant PaSOD-1 and -2 were examined. PaSOD, Cys-less CuZnSOD protein in *Paenibacillus lautus; PBS, phosphate-buffered saline*.

Figure 8*A* shows a representative growth curve of *P. lautus* that was incubated at 30 °C in an L-shaped test tube by using a rocking incubator at 50 rpm. Using our anti-PaSOD antibody, an intense band was observed around 30 kDa throughout the bacterial growth, whereas its mobility was slightly lower than that of recombinant PaSOD-1/2 (Fig. 8*B*). After the semipurification with the ammonium sulfate precipitation followed by the hydrophobic interaction chromatography (see the Experimental procedures section), the analysis with the protein sequencing revealed that the species of the intense band turned out to be SufC (28.9 kDa; N-terminal sequence, STKFV). Instead, at a late stage in the growth curve (>30 h), we noticed an anti–PaSOD-positive band that exhibited almost the same electrophoretic mobility with that of the recombinant PaSOD-2 protein (Fig. 8*B*). Again by the ammonium sulfate precipitation followed by the hydrophobic interaction chromatography, the endogenous species showing the same electrophoretic mobility with that of recombinant PaSOD-2 were semipurified (Fig. 8*C*, *left panel*). The semipurified sample was then separated by native-PAGE and probed by the SOD activity staining as well as the immunoblotting with anti-PaSOD antibody after electroblotted on a polyvinylidene difluoride (PVDF) membrane (Fig. 8*C*, *right panel*). In the fraction eluted with phosphate-buffered saline (PBS) from the hydrophobic interaction column, the achromatic band showing the SOD activity was detected at almost the same electrophoretic mobility with that of PaSOD-2 (Fig. 8*C*, *middle panel*) and was also found to be recognized by our anti-PaSOD antibody (Fig. 8*C*, *right panel*). While the expression of PaSOD-1 in *P. lautus* could not be clearly confirmed in the current growth condition, those results suggest that PaSOD-2 was expressed in *P. lautus* at a late growth phase.

## Discussion

In this study, we have shown that PaSOD-1/2 is composed of two distinct domains (*i.e.*, PaSOD-1/2^NTD^ and PaSOD-1/2^CTD^) and forms a homodimer in the unprecedented subunit configuration; namely, PaSOD-1^CTD^ structurally homologous to CuZnSOD is not involved in the dimerization, whereas PaSOD-1^NTD^ with a novel fold constitutes the dimer interface. The subunit configuration in PaSOD-1 could be compared with the homodimerization in a “domain-swapping” mechanism, where two or more identical monomers exchange structural elements and fold into dimers or multimers whose units are structurally similar to the original monomer (23, 24). Nonetheless, it is quite difficult to assume the PaSOD-1 monomeric structure resembling the interactions in the homodimer (Fig. 4), and the domain swapping mechanism would not be well fit for the description of the PaSOD homodimerization. As another comparable example, acyl aminoacyl peptidase from *Sporosarcina psychrophile* is known to form a homodimer in the “arm-exchange” mechanism, where the N-terminal helix at one subunit reciprocally inserts into the other subunit and behaves like an arm that allows the subunits to “hug” each other (25, 26). In the PaSOD-1 homodimer, PaSOD-1^NTD^ could be regarded as the arm (Fig. 4); unlike acyl aminoacyl peptidase, however, the arm of one subunit interacts with the arm of the other subunit. Therefore, we suggest that PaSOD-1 homodimerizes with each subunit shaking hands through PaSOD-1^NTD^ rather than hugging each other.

Together with the novel configuration of the subunits, PaSOD-1/2 is the first example of the active CuZnSOD without having the S–S bond. The absence of the S–S bond in PaSOD-1/2 is rather surprising to us, because the S–S bond conserved among all known CuZnSODs plays critical roles in the protein structure and function. Our crystal structural analysis has revealed that the S–S bond is substituted with hydrophobic and hydrogen-bonding interactions in PaSOD-1 for maintaining the catalytically competent conformation. The substitution of Ser for the disulfide-bonding Cys residues in yeast CuZnSOD is known to dramatically decrease the enzymatic activity (8); in contrast, a significant level (∼70%) of the activity remained when the Cys residues in hSOD1 were replaced with Ala (27). Although the Cys to Ala substitution in EcSodC nullified the activity (22), the hydrophobic interactions could partly replace the conserved S–S bond in CuZnSOD for the maintenance of the activity.

Nonetheless, the conformational stability of CuZnSOD was known to decrease by losing the S–S bond because of its reduction or the amino acid substitution. We have reported that hSOD1 misfolds to abnormal aggregates/oligomers upon either losing or shuffling the S–S bond, which is considered as a pathological process in a familial form of amyotrophic lateral sclerosis (28, 29, 30). Moreover, EcSodC was found to be easily degraded in the periplasmic space when the S–S bond was not introduced (22). These observations thus substantiate the need *in vivo* to regulate the introduction and maintenance of the S–S bond in CuZnSOD. Indeed, the conserved S–S bond in many of eukaryotic CuZnSOD is known to be introduced by a copper chaperone, CCS (8). In a proposed mechanism of the CCS-mediated maturation of CuZnSOD, the copper ion is supplied from CCS to the disulfide-reduced zinc-bound form of CuZnSOD, when the S–S bond is introduced into CuZnSOD likely through the copper-mediated sulfenylation at the Cys residue(s) (8, 31, 32). Once the S–S bond is formed, CuZnSOD strongly favors the homodimeric state (20), in which the S–S bond is occluded at the dimer interface; therefore, CuZnSOD maintains the S–S bond and thus remains active even in reducing environment of the cytosol.

Regarding bacterial CuZnSOD, it remains obscure how the S–S bond is introduced. In Gram-negative bacteria, CuZnSOD is localized in oxidizing environment of the periplasmic space (33), where the disulfide-introducing system (so called the Dsb system) is well characterized (34); in fact, a periplasmic disulfide oxidoreductase DsbA has been proposed to introduce the S–S bond in CuZnSOD albeit not requisite (22, 35). Also, the Dsb proteins can fix incorrectly introduced S–S bonds in periplasmic proteins by disulfide reduction/rearrangement (34). In contrast, Gram-positive bacteria, to which *Paenibacillaceae* belongs, have no traditional periplasmic space; the plasma membrane is surrounded by thick layers of peptidoglycan. PaSOD has the N-terminal signal peptide for the secretion but was not detected in the culture media (data not shown); therefore, we suppose that PaSOD-1/2 would be trapped in a little space between the plasma membrane and the peptidoglycan layer. Given the diffusive nature of peptidoglycan, the plasma membrane is considered to be exposed to the extracellular milieu, and secreted proteins with Cys residues would be susceptible to aberrant oxidation (36). Nonetheless, a disulfide-introducing/correcting system in Gram-positive bacteria (*Firmicutes*, in particular) has been considered not to play significant roles in the protein folding albeit less characterized, and some *Firmicutes* do not even express any *dsb*-like genes (36). Rather, *Firmicutes* are known to contain few proteins with Cys residues in their secreted proteomes and would thereby avoid the protein misfolding with aberrant S–S bonds (37). No Cys residue in PaSOD thus appears to be consistent with such a tactic of *Firmicutes*.

In the course of the BLAST search, nonetheless, we noticed that proteins homologous to PaSOD were not hit in *Paenibacillus polymyxa* and *Paenibacillus larvae*, the two common species of *Paenibacillus*. Indeed, when the genome of *P. polymyxa* and *P. larvae* was searched for proteins annotated as SOD, only Mn-containing (*i.e.*, SodA) but not Cu-containing (*i.e.*, PaSOD and CuZnSOD) SOD was found. We then searched proteins annotated as Cu-containing SOD in *Paenibacillus* in the NCBI database by using “Paenibacillus and Sod_Cu” as the keyword. Interestingly, some species of *Paenibacillus* such as *Paenibacillus aceris* were found to have canonical CuZnSOD with the disulfide-bonding Cys residues instead of PaSOD. Also, we noticed that the three species (*Paenibacillus mucilaginosus*, *Paenibacillus puerhi*, and *Paenibacillus* sp. Y412MC10) were equipped with both CuZnSOD and PaSOD. In terms of Cu-containing SOD, therefore, the species of *Paenibacillus* can be categorized into four classes: namely, the species with only PaSOD (*e.g.*, *P. lautus* examined in this study), only CuZnSOD (*e.g.*, *P. aceris*), neither CuZnSOD nor PaSOD (*e.g.*, *P. polymyxa*), and both CuZnSOD and PaSOD (*e.g.*, *P. mucilaginosus*). These results suggest that the activity of CuZnSOD is not essential to the growth of *Paenibacillus*. Indeed, the insignificant level of the PaSOD-1/2 expression would be consistent with no need of PaSOD-1/2 for the growth and/or survival of *P. lautus* in the current experimental conditions. Alternatively, the expression might be triggered in response to some stresses, but *P. lautus* was isolated from human intestinal tracts (dejecta of children) (38) and also from soil (39, 40), both of which are very ordinary environment for bacteria. Understanding the significance of PaSOD-1/2 thus awaits further investigations particularly on the physiology of *P. lautus*.

While both recombinant PaSOD-1 and -2 possessed the SOD activity, furthermore, it is interesting to note that several representatives in *Firmicutes* possess CuZnSOD-like proteins with no enzymatic activity; for example, *Bacillus subtilis* has a CuZnSOD-like protein (called YojM), but the copper-binding site of this protein is disrupted, resulting in no ability to bind a copper ion and hence no enzymatic activity (41). Another *Firmicute*, *Streptococcus pneumoniae*, also appears to have no active CuZnSOD (42), whereas “SOD (Cu–Zn) precursor” is registered in the database (*e.g.*, accession no.: CJG06664). This precursor is yet to be tested for the activity as CuZnSOD but lacks some of the amino acid residues in the copper- and zinc-binding site. Therefore, the CuZnSOD-like proteins in *Firmicutes*, which include PaSOD in *Paenibacillaceae*, might have an alternative but as-yet-unknown function(s); indeed, the functional significance of bacterial CuZnSOD remains ambiguous even in one of the most studied organism, *E. coli*.

In conclusion, we have identified a novel CuZnSOD protein, PaSOD, which has no Cys residues and thus lacks the conserved S–S bond. PaSOD is composed of the N-terminal dimerization domain with a novel fold and the C-terminal CuZnSOD domain with the enzyme activity. The conserved S–S bond in CuZnSOD plays an important role in maintaining the catalytically competent protein conformation and is replaced by hydrophobic and hydrogen-bonding interactions in PaSOD. While physiological roles of PaSOD remain obscure, Cys-less PaSOD in *Paenibacillaceae* will not need to form the S–S bond for the maintenance of the protein conformation and is hence considered to be functional irrespective of redox environment.

## Experimental procedures

### DNA cloning

*P. lautus* NBRC 15380 was obtained from NBRC and cultured in 2xYT media with trace elements (see later) at 30 °C. By following the method previously described (43), the genomic DNA of *P. lautus* was isolated. The genome sequence of *P. lautus* NBRC 15380 was already registered at NCBI (Fig. S2), based upon which primers covering the open reading frame of PaSOD-1 (WP_246059278.1) and PaSOD-2 (WP_127589926.1) were designed; more specifically, a pair of 5′-ATT CCA GAT TGC GTT GAG GAT AAA ATG GGG-3′ and 5′-GCC GAT AGA CAT TTT CCC AAT TCG TAA TTC-3′ (for WP_246059278.1) and also a pair of 5′-GAC GAT GTG TTG ATC GTG TTT GAA CTC GAC-3′ and 5′-CCT GTT CTG ATC CAA ACC GGA ACA TCG ATG-3′ (for WP_127589926.1) were prepared. Using those primers and the isolated genomic DNA, the PaSOD-1 and -2 genes were amplified with PCR using KOD FX neo (TOYOBO) and sequenced. In this study, the plasmids expressing PaSOD-1/2 and their variants (also see later) were prepared by using In-Fusion HD Cloning Kit (Clontech, Takara Bio, Inc).

### Protein preparation

The N-terminal region of PaSOD-1 and -2 (Met1–Ala49; amino acid numbering was based upon WP_127590094.1) is predicted to function as a signal peptide and to be probably cleaved in the mature form of PaSOD-1 and -2. We thus introduced a complementary DNA fragment corresponding to Asn50–Glu269 of PaSOD-1 and -2 into the multiple cloning site of a modified pET-15b plasmid vector with In-Fusion HD Cloning Kit (Clontech, Takara Bio, Inc), in which the thrombin cleavage site was replaced with the HRV3C cleavage site. PaSOD proteins were overexpressed in *E. coli* BL21(DE3) transformed with the plasmid. The *E. coli* cells were shaken-cultured in an LB medium with ampicillin at 37 °C until the absorbance at 600 nm reached around 0.6. Expression of PaSOD proteins was then induced with 0.5 mM isopropyl-1-thio-β-d-galactopyranoside at 20 °C overnight.

The supernatant obtained by centrifugation of cell lysates at 20,000*g* for 15 min was loaded on a cOmplete His-Tag Purification Column (1 ml, Roche). After washed with a buffer containing 50 mM Na–Pi and 500 mM NaCl at pH 7.0, PaSOD proteins were eluted from the column with a buffer containing 50 mM Na–Pi, 100 mM NaCl, and 100 mM imidazole at pH 7.0. An N-terminal His-tag of an eluted PaSOD protein was cleaved by incubation with His-tagged HRV3C protease at 4 °C overnight. The cleaved His-tag and the His-tagged HRV3C protease were removed by a cOmplete His-Tag Purification Column, and a tag-free mature PaSOD was further purified by SEC using a gel filtration column (Cosmosil 5Diol-300-II; Nacalai Tesque, Inc) equilibrated with a buffer containing 50 mM Mops and 100 mM NaCl at pH 7.0 (MN buffer). Concentration of PaSOD was spectroscopically determined from its absorbance at 280 nm using 12,950 M^−1^ cm^−1^ and as a molar extinction coefficient, whereas 11,460 M^−1^ cm^−1^ and 1490 M^−1^ cm^−1^ were used for PaSOD-1 with F179S mutation and PaSOD-1/2^CTD^ (Gly125–Glu269), respectively. Copper and zinc contents in the samples were checked with graphite furnace atomic absorption spectroscopy (AA-7000; Shimadzu).

### Characterization of PaSOD proteins

The activity assay was performed as described previously (17). The molecular size of PaSOD in solution was examined by online SEC–MALS. PaSOD and PaSOD^CTD^ (20 μM) in the MN buffer were loaded on a gel filtration column (Cosmosil 5Diol-300-II; Nacalai Tesque, Inc) fitted to an HPLC system (Shimadzu), and the absorbance change at 280 nm of the elution was monitored. The molecular size of the protein eluted from the column was determined by MALS using miniDAWN TREOS (WYATT Technology) connected on-line to the HPLC system.

### Analysis of endogenous PaSOD

A 5 ml culture of *P. lautus* NBRC 15380 cells in 2xYT medium supplemented with trace elements (0.1 mM FeCl_3_, 2 mM MgSO_4_, 0.05 mM MnCl_2_, 0.1 mM CaCl_2_, 1 μM CuCl_2_, 20 μM ZnCl_2_, 0.4 μM CoCl_2_, 0.4 μM NiCl_2_, 0.4 μM Na_2_MoO_4_, and 0.4 μM H_3_BO_3_) was prepared at 0.1 of the absorbance at 600 nm in an L-shaped test tube and then incubated at 30 °C using a rocking incubator (TVS062CA; ADVANTEC) at 50 rpm. The incubator can automatically monitor the absorbance of the cultures. Addition of the trace elements was found to significantly facilitate the cell proliferation. The cells collected by centrifugation at 3000*g* were resuspended in PBS containing 2% Triton X-100, 1 mM EDTA, and cOmplete Protease Inhibitor Cocktail and was lysed by sonication using BIORUPTOR II (Sonicbio Co Ltd). The total protein concentrations in the lysates were measured by Micro BCA Protein Assay Kit (Thermo Scientific). For the detection of PaSOD with Western blotting analysis, the lysates were prepared in the Laemmli sample buffer with 6.7% β-mercaptoethanol, separated in 12.5% polyacrylamide gels by SDS-PAGE, and then blotted on a PVDF membrane. After the membranes were blocked with 1% (w/v) skim milk in PBS containing 0.05% Tween-20, the blots were probed with a polyclonal antibody (anti-PaSOD) that was raised in rabbits immunized with a peptide corresponding to Gly246–Gly259 in PaSOD-1 with an additional Cys at its N terminus (Eurofins Genomics) and affinity-purified with the peptide conjugated with SulfoLink Coupling Resin (Thermo Fisher Scientific). The anti-PaSOD antibody was confirmed to recognize both PaSOD-1 and -2 by Western blotting analysis using the recombinant proteins.

To semipurify endogenous PaSOD proteins, *P. lautus* NBRC 15380 was first cultured in 500 ml of 2xYT media supplemented with the trace metals by shaking at 30 °C, 160 rpm for 5 days. The cells were then collected and lysed in PBS containing 2% Triton X-100, 5 mM MgSO_4_, and 7 μg/ml DNase I by ultrasonication. The supernatant was obtained by centrifugation at 20,000*g* for 15 min and purified by the ammonium sulfate precipitation. The precipitates in 70% saturation of ammonium sulfate were redissolved in PBS with 50% saturation of ammonium sulfate and loaded on HiTrap Phenyl FF (1 ml; Cytiva). After the column was washed with PBS containing 10% saturation of ammonium sulfate, the bound proteins were eluted first with PBS and then with water. The eluted fractions were analyzed by Western blotting with anti-PaSOD antibody; the bands corresponding to those recognized with the antibody were also electroblotted on the PVDF membrane, stained with Coomassie Brilliant Blue, and then analyzed by protein sequencing (Osaka University).

### In-gel SOD activity assay

The activity was examined by an in-gel assay following the separation of proteins with native-PAGE on ice using a 10% polyacrylamide gel (44). After the electrophoresis, the gel was soaked in 1 g/l nitro blue tetrazolium followed by 100 mM potassium phosphate at pH 7.0 containing 140 μM riboflavin 5′-phosphate sodium and 0.2% (v/v) *N*,*N*,*N*′,*N*′-tetramethyl ethylenediamine in the dark. The gel was then exposed to a light box until achromatic bands representing the SOD activity were developed.

### Differential scanning fluorometry

PaSOD-1 and PaSOD-1^CTD^ (final concentration at 75 μM) containing 75 μM CuSO_4_ and 75 μM ZnSO_4_ were mixed with SYPRO Orange Protein Gel Stain (final concentration at 50×) (Thermo Fisher Scientific) in 100 mM Na–Pi/100 mM NaCl/0.5 mM EDTA, pH 7.0. A Real-Time PCR Thermal Cycler snow (BMBio) was used to measure the fluorescence from 20 to 95 °C.

### Crystallization

All crystals were grown using the hanging-drop vapor-diffusion method at 20 °C. Recombinant PaSOD-1 at a concentration of 35 mg/ml was crystallized using 22.5% PEG 4000 as precipitant with 100 mM sodium acetate buffer at pH 5.0. The dimensions of the crystals with their spacegroup *P*2_1_2_1_2_1_ used for data collection at wavelength 1.275 Å and 1.373 Å were 2.0 mm × 1.0 mm × 0.05 mm and 0.4 mm × 0.2 mm × 0.2 mm, respectively. Recombinant PaSOD-2 at a concentration of 35 mg/ml was crystallized using 60% Tacsimate (pH 7.0).

### Data collection and processing

Crystals were soaked in a solution listed on the Well-D4 line on the MORPHEUS protein crystallization screen as a cryoprotectant before diffraction data collection. All diffraction data were collected at 100 K. The dataset at a wavelength close to the copper absorption edge (λ = 1.373 Å) was collected on the beamline AR-NW12A at Photon Factory using PILATUS3 S2M detector (Dectris). Also, the dataset at a wavelength close to the zinc absorption edge (λ = 1.275 Å) was collected on the beamline Xo6SA at Swiss Light Source using EIGER 16M X detector (Dectris). Diffraction data were processed by XDS (45) and then scaled using the program scala (46) in CCP4. Anomalous difference Fourier map was obtained by fast Fourier transform incorporated in CCP4. Data collection and processing statistics are listed in Table S1.

### Structure solution, refinement, and modeling

All PaSOD-1 structures were solved by single-wavelength anomalous diffraction method using the Crank2 program (47) with anomalous signals from either copper- or zinc ion–bound in PaSOD-1. Maximum likelihood refinement of these models was carried out using Refmac5, version 5.8, as implemented in CCP4, with 5% of random reflections set aside to calculate free *R*-factor values. Water molecules were gradually added to the model using the COOT program (48) and positioned when well-defined positive peaks were present in both 2*F*_o_−*F*_c_ and *F*_o_−*F*_c_ electron density maps and also when the water molecules formed hydrogen bonds with either protein atoms or other water molecules. Models were manually adjusted using COOT. Multiple conformations of side-chain and main-chain residues were accounted for in the last stages of refinement. The quality of the final model for each structure was assessed with MolProbity (49). Refinement and modeling parameters are summarized in Table S1. The coordinates of the PaSOD-1 structure obtained by the analysis of the dataset at a wavelength of 1.275 Å have been deposited in the Protein Data Bank under the accession code, 8IMD.

For PaSOD-2 crystal, the initial phase was calculated by the molecular replacement method using the structure model of PaSOD1 as a search model using PHASER (50) in the CCP4 suite. An initial log-likelihood gain value of 350 and a translation function Z-score of 15.5 indicated that the correct solution has been found. The obtained phases, however, yielded a poor electron density map that was not sufficient for model building and structure refinement.

## Data availability

The data that support the findings in this study are available upon request.

## Supporting information

This article contains supporting information (51).

## Conflict of interest

The authors declare that they have no conflicts of interest with the contents of this article.
