# Supplementary material for: Characterization of a novel cysteine-less Cu/Zn-superoxide dismutase in Paenibacillus lautus missing a conserved disulfide bond
Source: J Biol Chem. 2023 Jul 11;299(8):105040. doi: 10.1016/j.jbc.2023.105040 (PMC10432803; doi:10.1016/j.jbc.2023.105040)
Supplement: Supporting Table S1 and Figures S1–S4 [file mmc1.pdf]

Table S1 Data collection, reduction and refinement statistics on the analysis of PaSOD-1 crystals

	**Zn absorption edge**	**Cu absorption edge**
**Data collection and reduction**		
Wavelength (Å)	1.275	1.373
Space group	$P2_12_12_1$	$P2_12_12_1$
Cell dimensions (Å)	a = 45.24, b = 53.61, c = 169.57	a = 45.30, b = 53.44, c = 170.26
Resolution limits (Å)	45.31-1.45 (1.53-1.45)	45.26-1.50 (1.53-1.50)
No. reflections	1,370,517 (168,427)	831,710 (40,169)
No. unique reflections	74,112 (10,562)	64,043 (2,998)
Completeness (%)	99.8 (98.9)	95.6 (90.5)
Multiplicity	18.5 (15.9)	13.0 (13.4)
$I/\sigma(I)$	26.6 (1.8)	23.7 (2.5)
R_{merge} (%)	5.4 (153.8)	5.7 (99.4)
CC1/2	1.000 (0.647)	0.999 (0.811)
Wilson B-factor (Å^2)	18.3	19.4
**Refinement**		
No. protein atoms	3,143	3,107
No. solvent atoms	307	245
No. copper ions	2	2
No. zinc ions	2	2
No. MES molecules	2	1
R_{cryst} (%)	18.82	20.14
R_{free} (%)	22.06	23.77
ML-based ESU (Å)	0.056	0.066
B_{av} main-chain (Å^2)	23.987 (Chain A)<br>26.301 (Chain B)	25.223 (Chain A)<br>30.583 (Chain B)
r.m.s. deviations from ideal		
Bond length (Å)	0.0123	0.0121
Bond angles (deg.)	1.76	1.78

Values in parentheses are for the highest resolution shell.

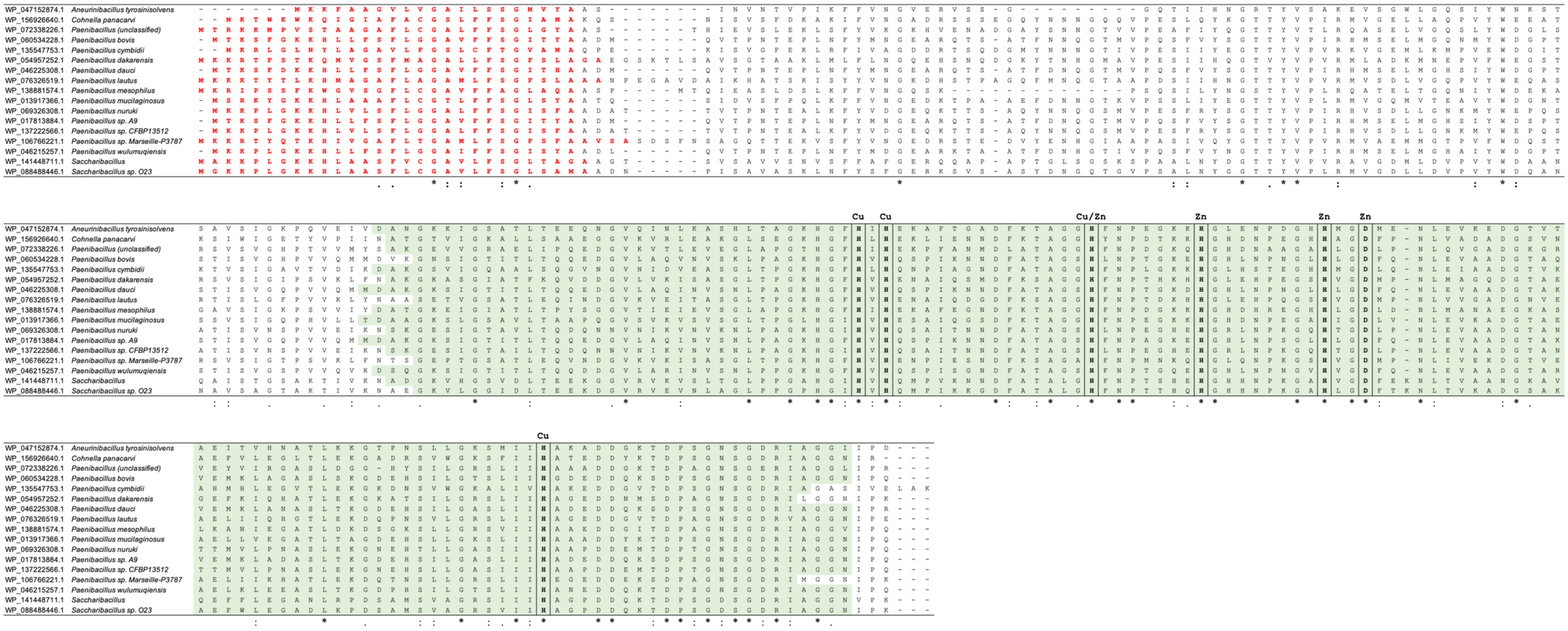

Figure S1 **Multiple alignment of PaSOD amino acid sequences in a family of *Paenibacillaceae*** The 17 reference sequences registered as "Cu_amine_oxidN1 and Cu-Zn_Superoxide Dismutase domain-containing protein" in the NCBI database were aligned. All of those sequences are composed of the N-terminal signal sequence (red) followed by the copper amine oxidase N-terminal domain and the CuZnSOD domain (green). The residues involved in the copper and zinc binding are conserved, and no Cys residues are found in the matured form of the proteins.

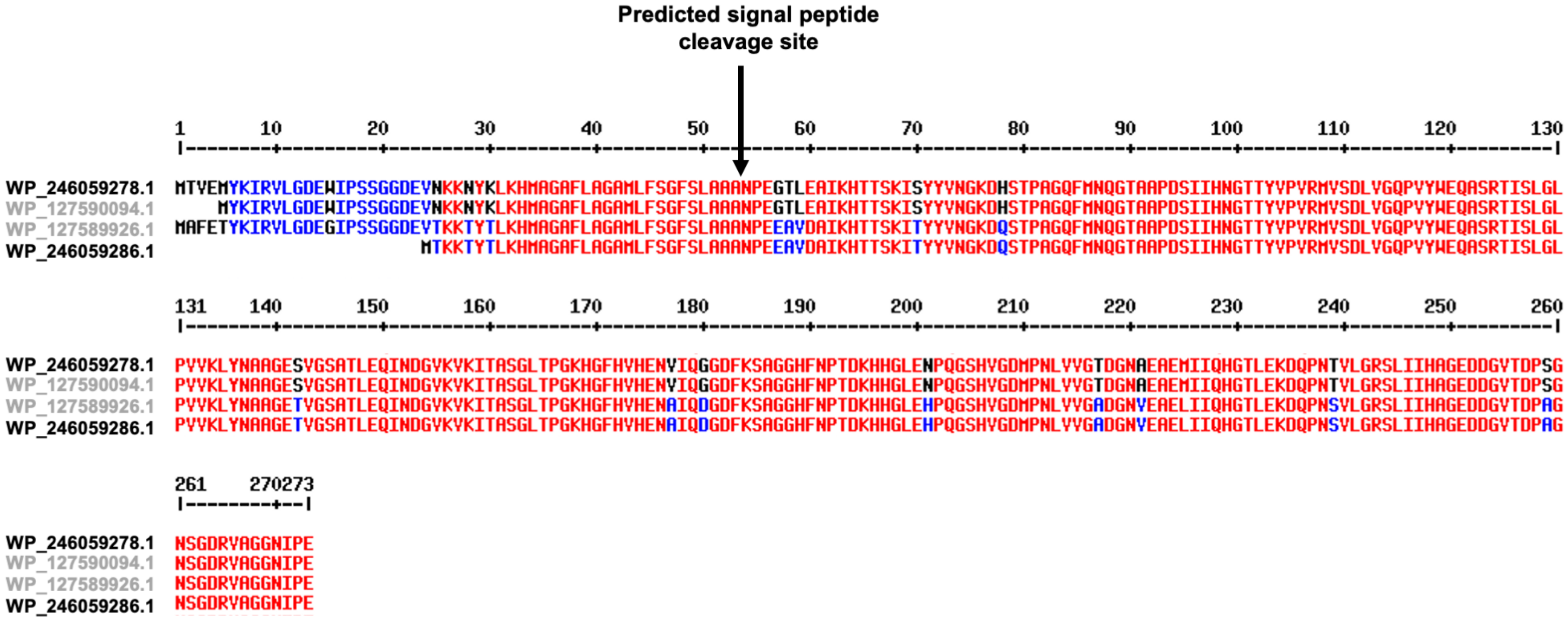

Figure S2 Multiple alignment of PaSOD amino acid sequences in *P. lautus* NBRC 15380 In the database provided by NCBI, two sequences colored by gray (i.e. WP_127590094.1 and WP_127589926.1) were registered as a superoxide dismutase family protein but are now obsolete and replaced with the sequences, WP_246059278.1 and WP_246059286.1. The obsolete and renewed sequences are different only in the start site. Because the N-terminal region will be cleaved as a signal peptide, the amino acid sequence of PaSOD-1/2 in the matured form is the same between the obsolete and renewed sequences. The alignment was performed by MultAlin (Corpet, F. *Nucleic Acids Res* **1988** 16 10881).

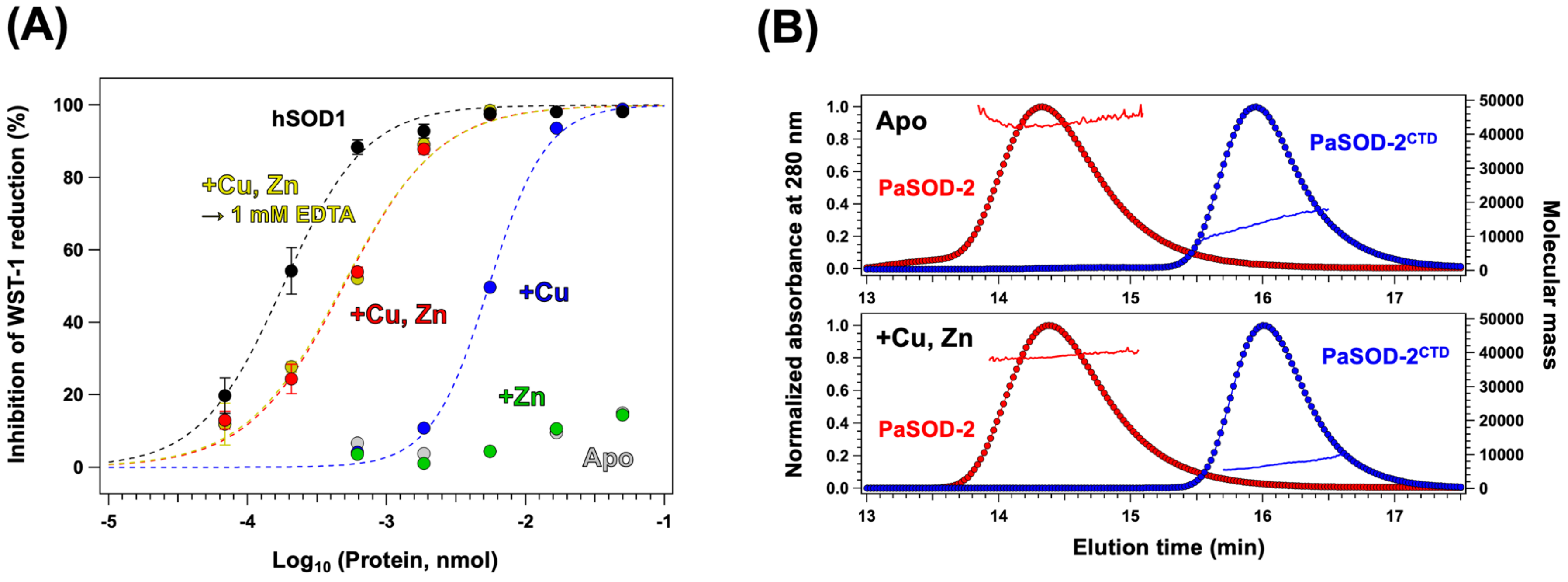

PaSOD-2 (+Cu, Zn) : IC_{50} = 0.49 ± 0.05 pmol

PaSOD-2 (+Cu, Zn) + 1 mM EDTA : IC_{50} = 0.48 ± 0.02 pmol

Figure S3 **PaSOD-2 exhibits enzymatic activity as CuZnSOD** **(A)** Activity assay was performed using (gray) as-isolated apo form of PaSOD-2, PaSOD-2 with an equimolar amount of either (green) Zn^{2+} or (blue) Cu^{2+}, and (red) PaSOD-2 with an equimolar amount of both Cu^{2+} and Zn^{2+}. Following addition of an equimolar amount of both Cu^{2+} and Zn^{2+}, PaSOD-2 was further treated with 1 mM EDTA, and the activity assay was performed (yellow). As a positive control, the activity of human CuZnSOD in the holo form was also assayed (black). The activity values (IC_{50}) of PaSOD-2 before and after the addition of 1 mM EDTA were also shown. **(B)** Size-exclusion chromatograms of PaSOD-2 proteins (20 μM in the MN buffer), which were monitored at 280 nm and normalized for comparison (left axis), were shown with the molecular mass estimated by MALS (right axis): the upper panel, PaSOD-2 (red) and PaSOD-2^{CTD} (blue) in the apo form: the lower panel, PaSOD-2 (red) and PaSOD-2^{CTD} (blue) in the holo form.

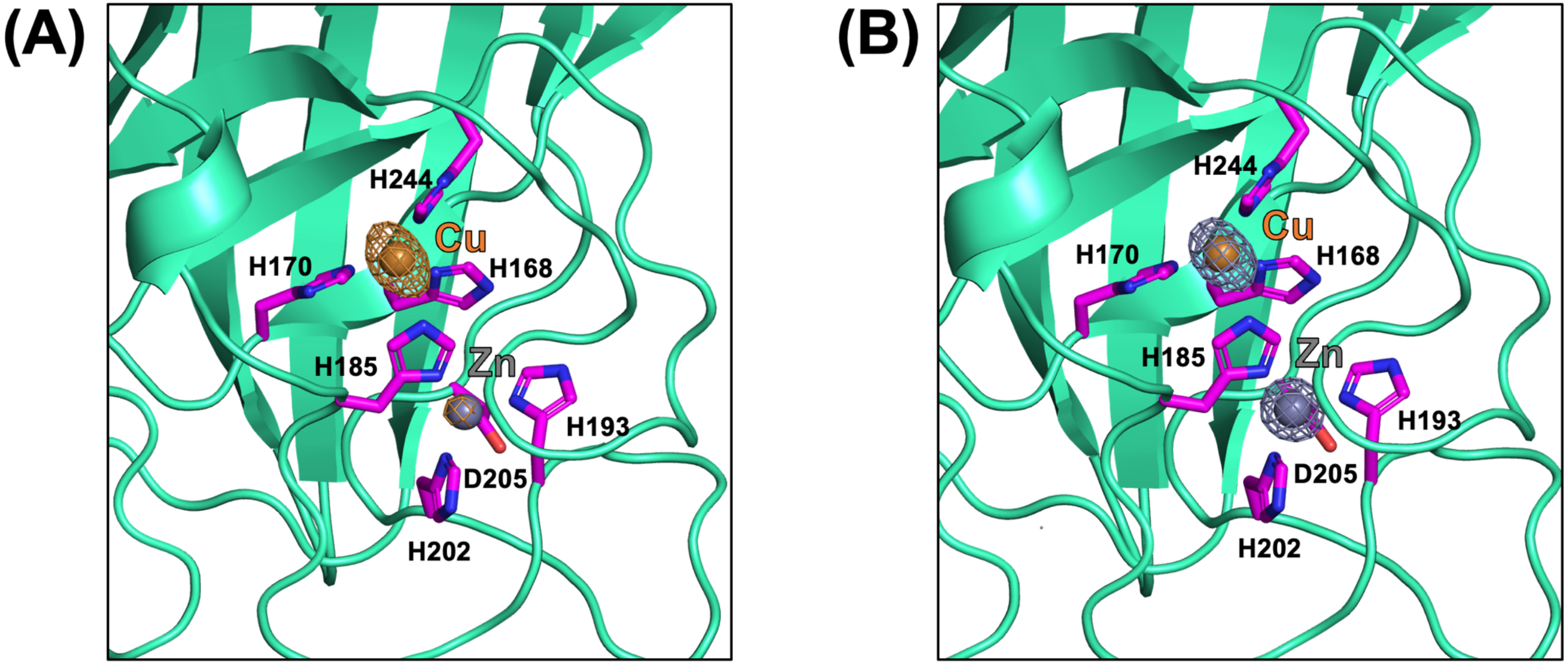

Figure S4 **Characterization of the binding metals at the distinct metal-binding sites in PaSOD-1** Using diffraction data measured at wavelengths of **(A)** 1.373 Å and **(B)** 1.275 Å, anomalous scattering difference maps around the metal-binding sites in PaSOD-1 were calculated at the 5σ level and represented as mesh. Cu and Zn atoms are represented as spheres, and the ligands for binding of the metal ions are shown in a stick model. The difference density was observed at both copper- and zinc-binding sites in PaSOD-1 when measured at wavelength of 1.275 Å, where anomalous scattering from both copper and zinc occurs. In contrast, the measurement at wavelength of 1.373 Å, where anomalous scattering from copper but not zinc occurs, resulted in significant decrease of the difference density at the zinc-binding site. These observations are consistent with our assignment of a copper and zinc ion at the distinct metal-binding sites as indicated in the figure.
